# Combined Metabonomic and Quantitative RT-PCR Analyses Revealed Metabolic Reprogramming Associated with *Fusarium graminearum* Resistance in Transgenic *Arabidopsis thaliana*

**DOI:** 10.3389/fpls.2017.02177

**Published:** 2018-01-04

**Authors:** Fangfang Chen, Caixiang Liu, Jingtao Zhang, Hehua Lei, He-Ping Li, Yu-Cai Liao, Huiru Tang

**Affiliations:** ^1^CAS Key Laboratory of Plant Germplasm Enhancement and Specialty Agriculture, Wuhan Botanical Garden, Chinese Academy of Sciences, Wuhan, China; ^2^College of Plant Science and Technology and College of Life Science and Technology, Huazhong Agricultural University, Wuhan, China; ^3^CAS Key Laboratory of Magnetic Resonance in Biological Systems, State Key Laboratory of Magnetic Resonance and Atomic and Molecular Physics, National Centre for Magnetic Resonance in Wuhan, Wuhan Institute of Physics and Mathematics, Chinese Academy of Sciences, Wuhan, China; ^4^State Key Laboratory of Genetic Engineering, Zhongshan Hospital and School of Life Sciences, Collaborative Innovation Centre for Genetics and Development, Shanghai International Centre for Molecular Phenomics, Metabonomics and Systems Biology Laboratory, Fudan University, Shanghai, China

**Keywords:** *Arabidopsis thaliana*, *Fusarium graminearum*, metabolic responses, mycotoxins, Chi-CWP2, NMR

## Abstract

Fusarium head blight disease resulting from *Fusarium graminearum* (FG) infection causes huge losses in global production of cereals and development of FG-resistant plants is urgently needed. To understand biochemistry mechanisms for FG resistance, here, we have systematically investigated the plant metabolomic phenotypes associated with FG resistance for transgenic *Arabidopsis thaliana* expressing a class-I chitinase (Chi), a *Fusarium*-specific recombinant antibody gene (CWP2) and fused Chi-CWP2. Plant disease indices, mycotoxin levels, metabonomic characteristics, and expression levels of several key genes were measured together with their correlations. We found that *A. thaliana* expressing Chi-CWP2 showed higher FG resistance with much lower disease indices and mycotoxin levels than the wild-type and the plants expressing Chi or CWP2 alone. The combined metabonomic and quantitative RT-PCR analyses revealed that such FG-resistance was closely associated with the promoted biosynthesis of secondary metabolites (phenylpropanoids, alkanoids) and organic osmolytes (proline, betaine, glucose, *myo*-inositol) together with enhanced TCA cycle and GABA shunt. These suggest that the concurrently enhanced biosyntheses of the shikimate-mediated secondary metabolites and organic osmolytes be an important strategy for *A. thaliana* to develop and improve FG resistance. These findings provide essential biochemical information related to FG resistance which is important for developing FG-resistant cereals.

## Introduction

Fusarium head blight (FHB) resulting from *Fusarium graminearum* infection is a serious plant disease causing losses of several millions of tons cereals in worldwide production of wheat, barley and maize (Xu et al., 2005; Xu and Nicholson, 2009; Kazan et al., 2012; Zhang et al., 2013). Such losses include both FHB-caused reductions in crop yields and in usability of cereal grains contaminated by potent trichothecene mycotoxins of FG (Li et al., 2002; Kazan et al., 2012). FG-resistant cultivars is thus urgently required to overcome the problems although natural germplasm resources with *Fusarium* resistance are unavailable at present (Liu, 2001). Currently, FG control relies largely on the use of chemical fungicides. This often causes undesirable environmental consequences and the development of fungicide-resistant *Fusarium* populations (D'Mello et al., 2000; Leonard and Bushnell, 2003; Zhang et al., 2009). Therefore, the development of resistant plants using transgenic technology becomes a promising and environmentally friendly way for FHB control (Collinge et al., 2008, 2010). Such technology will also be beneficial to reduce or eliminate mycotoxins in cereals (Chen et al., 1999; Anand et al., 2003; Makandar et al., 2006).

In fact, progresses have been made in transgenic plants with FG resistance already. There are many studies aiming to enhance plant resistance against FG using transgenic approaches (Koch et al., 2013; Collinge et al., 2016; Majumdar et al., 2017). For example, expressing dsRNA to silence CYP51 genes of fungi could render susceptible plants highly resistant to FG in Arabidopsis and barley (Koch et al., 2013). In addition, overexpressing the defense response genes α-1-purothionin, thaumatin-like protein 1 tlp-1, and β-1, 3-glucanase (Mackintosh et al., 2007) or expressing the maize b-32 antifungal gene (Balconi et al., 2007) in wheat could enhance resistance to FG. Furthermore, numerous antibodies derived from animals recognizing pathogen-specific antigens have been successfully transformed into plants (Safarnejad et al., 2011) to alter immune regulations and to enhance disease resistance of the resultant plants (Li et al., 2008; Safarnejad et al., 2009; Cervera et al., 2010). Some functional antibodies have also been derived from plants and already employed in commercial production (Schillberg et al., 2001). For instance, some genes encoding antifungal peptides from both plants and animals were transformed into Bobwhite, a model wheat cultivar, to improve FHB resistance (Chen et al., 1999; Anand et al., 2003; Makandar et al., 2006). Transgenic wheat with a gene encoding an antimicrobial peptide RsAFP2 showed improved resistance to fungal pathogens (Jha and Chattoo, 2010; Li et al., 2011).

Recently, the use of a chicken-derived *Fusarium*-specific recombinant antibody (CWP2) recognizing a surface antigen of *F. graminearum* attracted attention for developing the FG-resistant plants. When transforming CWP2 antibody that was fused to antifungal peptides into *A. thaliana* and wheat, both transgenic plants showed increased resistance to *Fusarium* pathogens and mycotoxins (Peschen et al., 2004; Li et al., 2008; Cheng et al., 2015). Such fusion proteins not only enhanced the stability of scFvs (Wörn et al., 2000), but also increased the activity of antimicrobial peptides and inhibitory effects on pathogens even with low level expression (Peschen et al., 2004). Such disease resistance plants improved both crop yields and quality. However, the biochemistry details of plants related to the disease-resistant functions of these fusion proteins remain to be fully understood and plant metabolic reprogramming is probably important.

Previous works have already shown metabonomics as a potentially powerful tool for obtaining the detailed metabolic responses of plants toward both biotic and abiotic stressors (Choi et al., 2004; Browne and Brindle, 2007; Paranidharan et al., 2008; Allen et al., 2009; Bollina et al., 2010; Dai et al., 2010b; Liu et al., 2010, 2017; Zhang et al., 2011; Sanchez et al., 2012; Kumar et al., 2016; Glaubitz et al., 2017). Tyrene-acrylic esters and indole-related alkaloids were found to be the main metabolites of *Catharanthus roseus* (L.) differentiating healthy leaves from the ones infected by ten different phytoplasma organisms (Choi et al., 2004). Metabonomic analysis of wheat leaves and stem tissues indicated that the levels of betaine, sucrose, glucose, glutamate, glutamine, alanine, *trans*-aconitic acid and some aromatic compounds were positively correlated with FHB resistance (Browne and Brindle, 2007). Metabolomic phenotypes have also been reported for numerous plant-pathogen interactions including *Brachypodium distachyon* and *Magnaporthe grisea* (William Allwood et al., 2006), mulberry leaf and phytoplasma (Gai et al., 2014), BPH infestation of rice (Liu et al., 2010, 2017), FG infected barley (Bollina et al., 2010), and wheat (Browne and Brindle, 2007; Paranidharan et al., 2008) together with chick pea (Kumar et al., 2016).

In this work, we investigated the FG resistance properties of *A. thaliana* lines expressing a wheat class I chitinase (Chi), a chicken-derived Fusarium-specific recombinant antibody (CWP2), a fusion protein (Chi-CWP2), and the wild type, respectively. We analyzed plant morphological phenotypes for disease indices, production of mycotoxins, metabonomic phenotypes together with the expressions of relevant genes in these *A. thaliana* plants with and without FG infection. The aim of this study was to reveal the metabolomic details of *A. thaliana* related to its FG resistance.

## Materials and methods

### Chemicals

Methanol, K_2_HPO_4_·3H_2_O and NaH_2_PO_4_·2H_2_O (all of analytical grade) were purchased from Guoyao Chemical Co. Ltd. (Shanghai, China). Both D_2_O (99.9% D) and sodium 3-trimethlysilyl [2, 2, 3, 3-^2^H_4_] propionate (TSP) were obtained from Cambridge Isotope Laboratories (Miami, USA). Mycosep 227^@^ were purchased from Romer Labs (USA). DON, 3Ac-DON, 15Ac-DON, TMSI, and TMCS were purchased from Sigma (USA). Acetonitrile was purchased from Thermo (USA).

### Plant materials

Four lines of *Arabidopsis thaliana* used in this study included wild-type (Columbia ecotype) and three transgenic lines expressing Chi, CWP2, and Chi-CWP2, all of which were generated via *Agrobacterium tumefaciens*-mediated transformation (Peschen et al., 2004). All *A. thaliana* seeds were surface-sterilized with 70% ethanol for 20 min, followed by 3 washes with 95% ethanol for 1 min and air-dried on sterilized filter paper. These seeds were then sown in the sterilized 0.5 × Murashige and Skoog medium containing 3% (w/v) sucrose and 7.5% agar (w/v). After germination, seedlings were transplanted to sterilized soil and grew for 2 weeks with a 16-h photoperiod at 22°C, 8,000 lux and 75% humidity. Nutrient solution was applied twice a week as previously described (Chen et al., 2000).

### Fungus culture

*F. graminearum* strain 5035 isolated from a scabby wheat spike in Wuhan, China, is a DON producer and highly pathogenic to wheat (Xu et al., 2010). This *Fusarium* strain was inoculated on sterilized glass-membrane paper over potato-dextrose agar at 28°C for 2 days and then cultured in CMC broth at 28°C (200 rpm) for 5 days. Conidiaspores were collected to prepare a suspension with concentration of 1 × 10^5^ spores per milliliter and stored at −20°C until used in subsequent inoculations.

### *F. graminearum* inoculation

The flowers of *A. thaliana* plants (~6-week-old) were carefully sprayed with a spore suspension containing 1 × 10^5^ spores per mL as done previously (Urban et al., 2002). Control plants were inoculated in the same manner but with deionized water instead of the spore suspension. The inoculated plants were kept in a large plastic propagator at 100% relative humidity for 7 days. During the first 2 days, lights were switched off to maintain darkness and turned on the third day. A numerical scoring method was applied to quantify the disease index of *A. thaliana* plants from flower infection, as previously described (Urban et al., 2002). After inoculation for 4 days, aerial materials of *A. thaliana* were respectively collected, frozen with liquid nitrogen, ground into powder and then stored at −80°C. Each sample was divided into two parts for metabonomic analysis and RNA extraction, respectively. Six biological replicates were sampled for each line. Matured seeds were also collected from new and old siliques for observation. The disease index was calculated from aerial mycelia development in flower, new siliques, and old siliques.

### Measurements of mycotoxins

The infected *A. thaliana* floral tissues from each plant line were harvested on day 7 post inoculation. All samples were stored at −80°C prior to analysis. The samples were then ground into fine powders with liquid nitrogen and dried in an electric blast drying oven, respectively. About 1 g such powder for each sample was added with 4 mL of acetonitrile-water solution (84:16, v/v) in a flask followed with shaking for 1 h. The resultant extract for each sample was individually obtained after removal of residues through vacuum filtration using a Buchner funnel. The crude extract solution was then purified with Mycosep 227^@^ columns. Mycotoxins were collected by eluating the columns with 5 mL of acetonitrile-water solution (84:16, v/v) and then evaporated to dryness. Group B trichothecenes (DON, 3Ac-DON, 15Ac-DON) were derivatized using 100 μL of trimethylsilyl imidazole (TMSI) containing 1% trimethylchlorosilane (TMCS) (v/v) for 15 min. Then 1 mL of isooctane (with 4 mg/L Mirex) was added and the reaction was then quenched with 1 mL of double distilled water. Upper layer was employed for analysis of mycotoxins using gas chromatography equipped with a mass spectrometer (QP2010, SHIMADZU) using a DB-5MS (30 m × 0.25 mm × 0.25 μm) capillary column as previously described (Chen et al., 2011). Each sample was analyzed twice with one in full scan mode (*m*/*z* 100–600) for the identification and the other in selected ion-monitoring (SIM) mode for quantifying the targeted analytes using known mycotoxin standards. Three independent replicates were analyzed for each plant line.

### Extraction of plant metabolites

Each freeze-dried plant powder sample (ca. 25 mg) from the aforementioned four plant lines in a microtube was added with 1 mL of pre-cooled 50% aqueous methanol (−40°C). These samples were then treated with intermittent sonication (i.e., 30 s of sonication with intermittent 30 s break) for 10 min in an ice bath. Following centrifugation (16,000 *g*, 4°C) for 10 min, the supernatant was transferred into a new microtube (5 mL); the remaining solid residues were extracted two more times using the same procedure and three resultant supernatants were combined. After removal of methanol under vacuum using a Speed-Vac Concentrator (Thermo SAVANT, SC110A-230), the supernatants were lyophilized in a freeze-drier for 24 h. The dried extracts were re-dissolved into 550 μL of phosphate buffer (0.1 M, pH 7.4) containing 10% D_2_O (v/v) and 0.02 mM TSP. After another centrifugation for 10 min, 500 μL of supernatant for each sample was transferred into a 5 mm NMR tube for NMR analysis. In the experiment, toxins and organic solvents were used in strict accordance with the local biological experimental safety management regulations, waste generated during the experiment were disposed by specialized agencies.

### NMR measurements

All NMR spectra were recorded at 298 K on a Bruker AV III 600 NMR spectrometer with a cryogenic TXI probe (Bruker Biospin, Germany) operating at 600.13 MHz for ^1^H. ^1^H NMR spectra were acquired using a standard one-dimensional NOESY-based pulse sequence (RD–90°–t_1_–90°–t_m_–90°–acquisition) with water signal suppressed during the recycle delay (RD, 2 s) and mixing time (t_m_, 100 ms). The 90° pulse length was about 9.5 μs and t_1_ was 3 μs. Sixty-four transients were collected into 32 k data points for each spectrum with a spectral width of 12 kHz. An exponential window function with line broadening factor of 0.5 Hz was applied to all free induction decays (FIDs) prior to Fourier transformation (FT). The chemical shifts were referenced to TSP at δ 0.00. For metabolite signal assignments, a set of 2D NMR (including ^1^H-^1^H COSY, ^1^H-^1^H TOCSY, ^1^H-JRES, ^1^H-^13^C HSQC, and ^1^H-^13^C HMBC) spectra were recorded and processed as previously reported (Teague et al., 2004; Xiao et al., 2008).

### Spectral processing and data analysis

After phase- and baseline-corrections using TOPSPIN (v3.1, Bruker Biospin GmbH, Germany), the region δ 0.50–9.50 of all ^1^H NMR spectra was divided into bins with a width of 0.003 ppm (1.8 Hz) using the AMIX software (v 3.8.3, Bruker Biospin GmbH, Germany). The region δ 4.40–5.15 was discarded to eliminate the effects of imperfect water pre-saturation. The spectral integrals of all buckets were then normalized to the dry weight of *A. thaliana* plants to give dataset in the form of signal area (metabolite quantity) per gram dry plant tissue. All NMR spectral bins were then subjected to the Student's test or nonparametric tests where appropriate with MUDA (multiple univariate data analysis) method as previously described (Duan et al., 2013). The results for inter-group differences can be displayed as a differential metabogram plot where *p*-values are color-coded for all variables with those metabolites having significant inter-group differences (*p* < 0.05) colored in red (Duan et al., 2013).

Concentration of some metabolites were calculated from the integrals of their NMR signals (non-overlapping ones) against that of an internal reference (TSP) with known concentration (Table 1). This is done by taking relaxation times (*T*_1_) into consideration (Table S1) as previously described (Dai et al., 2010a,b). The resultant data were subjected to statistical analyses (one way-ANOVA) using SAS software (V8, Statistics Analysis System, USA).

**Table 1:** Metabolite contents for wild type (WT) and transgenic *A. thaliana* expressing Chi, CWP2, and Chi-CWP2.

**Metabolites**	**WT*^a^***	**Chi*^a^***	**CWP2*^a^***	**Chi-CWP2*^a^***	**WT*^b^***	**Chi*^b^***	**CWP2*^b^***	**Chi-CWP2*^b^***
**SUGARS**								
Fructose	3.17 ± 0.72^c^	4.06 ± 1.06	3.61 ± 0.89	4.06 ± 0.45	3.83 ± 0.78	4.83 ± 0.89	4.61 ± 0.83	4.28 ± 0.50
*myo*-inositol	3.94 ± 0.50	2.89 ± 0.61^*^	3.61 ± 0.67	5.44 ± 0.67	3.72 ± 0.67	3.78 ± 0.67	4.50 ± 0.39^*^	5.11 ± 0.44^*^
α-glucose	21.83 ± 4.28	30.67 ± 3.72^*^	21.33 ± 2.11	26.28 ± 1.83	22.39 ± 1.61	23.72 ± 2.33	31.28 ± 2.22^*^	33.06 ± 1.83^*^
α-galactose	8.11 ± 2.94	7.44 ± 1.94	7.22 ± 2.39	8.94 ± 2.17	9.22 ± 1.28	9.83 ± 2.17	10.56 ± 2.17	10.00 ± 1.00
Sucrose	0.18 ± 0.06	0.20 ± 0.06	0.28 ± 0.06	0.28 ± 0.06	0.22 ± 0.06	0.27 ± 0.06	0.33 ± 0.11	0.33 ± 0.06
**AMINO ACIDS**								
Isoleucine	4.73 ± 0.92	5.04 ± 1.37	5.88 ± 1.07	5.73 ± 0.61	6.79 ± 0.53	7.33 ± 1.68	7.18 ± 0.92	7.33 ± 1.68
Valine	6.75 ± 1.20	6.67 ± 1.71	8.12 ± 1.54	8.55 ± 0.85	9.32 ± 0.77	10.00 ± 2.22	9.57 ± 1.45	10.17 ± 1.79
Threonine	14.37 ± 1.93	14.62 ± 2.77	14.71 ± 0.92	16.05 ± 2.10	17.48 ± 1.09	19.08 ± 2.61	21.18 ± 2.02^*^	16.47 ± 2.10
Alanine	11.96 ± 2.36	14.88 ± 4.04	14.16 ± 2.25	12.06 ± 1.35	22.36 ± 1.69	17.65 ± 4.49	18.87 ± 2.81^*^	15.06 ± 2.36^*^
Proline	8.66 ± 1.53	10.88 ± 1.71	10.35 ± 2.66	10.56 ± 1.05	10.39 ± 1.88	12.02 ± 1.88	12.45 ± 1.76^*^	12.87 ± 1.23^*^
Glutamine	32.53 ± 4.86	32.88 ± 5.00	36.30 ± 6.10	36.58 ± 4.04	35.34 ± 1.92	36.58 ± 5.68	41.10 ± 4.52	37.53 ± 4.32
Aspartate	11.88 ± 1.73	9.40 ± 2.41	10.68 ± 1.20	12.04 ± 0.90	15.11 ± 1.50	15.86 ± 1.80	14.74 ± 1.43	16.52 ± 2.26
Asparagine	49.4 ± 6.99	50.30 ± 3.03	62.88 ± 10.00^*^	83.18 ± 7.65^*^	71.59 ± 6.06	75.45 ± 7.58	75.38 ± 7.20	82.80 ± 4.47^*^
Phenylalanine	5.04 ± 1.35	6.24 ± 1.27	6.61 ± 1.03	10.85 ± 1.58^*^	5.82 ± 0.55	7.76 ± 1.33^*^	8.12 ± 0.61^*^	12.18 ± 0.97^*^
Trytophan	3.43 ± 0.44	3.09 ± 0.39	3.53 ± 0.44	4.56 ± 0.34^*^	3.58 ± 0.34	4.36 ± 0.49	4.07 ± 0.44	5.15 ± 0.49^*^
Tyrosine	1.63 ± 0.27	1.38 ± 0.37	1.73 ± 0.20	2.13 ± 0.18	1.80 ± 0.12	1.88 ± 0.38	1.92 ± 0.21	2.51 ± 0.23^*^
Histidine	1.61 ± 0.19	0.71 ± 0.19^**^	1.48 ± 0.13	2.06 ± 0.19^*^	1.55 ± 0.06	1.74 ± 0.19	1.87 ± 0.13	2.26 ± 0.13^*^
γ-aminobutyrate	11.46 ± 1.84	12.72 ± 0.78	12.08 ± 2.62	12.72 ± 1.17	16.99 ± 1.17	14.56 ± 1.55^*^	13.69 ± 1.36^*^	13.50 ± 1.36^*^
**ORGANIC ACIDS**								
Pyruvate	8.07 ± 1.83	8.58 ± 1.82	7.91 ± 0.50	9.27 ± 1.04^*^	10.71 ± 1.49	11.60 ± 2.63	11.82 ± 1.98	12.34 ± 1.62^*^
Succinate	5.66 ± 0.68	5.59 ± 0.85	6.36 ± 0.85	5.59 ± 0.34	5.85 ± 0.59	6.53 ± 0.93	6.69 ± 0.68	7.12 ± 0.93^*^
Malate	13.51 ± 1.12	14.16 ± 1.27	14.33 ± 1.04	15.90 ± 1.42	16.64 ± 1.49	22.09 ± 1.57^*^	20.45 ± 1.27^*^	24.48 ± 1.87^*^
Citrate	26.61 ± 2.66	24.48 ± 2.19	30.73 ± 3.28	39.48 ± 3.49^*^	18.02 ± 1.88	30.99 ± 2.40^*^	28.39 ± 2.24^*^	38.96 ± 5.10^*^
Fumarate	6.38 ± 0.43	8.45 ± 0.69^*^	7.50 ± 0.52^*^	5.86 ± 0.52	5.78 ± 0.69	6.21 ± 0.60	6.90 ± 0.60	7.41 ± 0.60^*^
**NUCLEOSIDE/TIDES**								
Uridine	2.42 ± 0.82	2.46 ± 0.94	2.17 ± 0.45	2.05 ± 0.53	2.54 ± 0.25	2.87 ± 1.15	2.87 ± 0.57	3.07 ± 0.53^*^
Adenosine	2.85 ± 0.90	2.85 ± 1.61	2.85 ± 0.82	3.11 ± 1.01	2.88 ± 0.97	3.18 ± 0.90	3.18 ± 0.56	3.52 ± 0.75
Inosine	0.11 ± 0.04	0.11 ± 0.04	0.11 ± 0.04	0.15 ± 0.04	0.11 ± 0.04	0.12 ± 0.04	0.13 ± 0.04	0.15 ± 0.04
Hypoxanthine	0.37 ± 0.07	0.37 ± 0.07	0.37 ± 0.07	0.37 ± 0.07	0.29 ± 0.07	0.33 ± 0.07	0.33 ± 0.07	0.35 ± 0.07
**CHOLINE METABOLITES**								
Choline	9.17 ± 0.83	10.32 ± 1.16	9.83 ± 0.99	9.74 ± 0.66	12.55 ± 0.83	11.73 ± 1.07	11.65 ± 0.53	10.16 ± 0.66^*^
Betaine	5.79 ± 0.82	6.14 ± 0.77^*^	5.46 ± 0.79	7.53 ± 0.70	6.82 ± 0.68	7.44 ± 0.97	7.60 ± 0.43^*^	9.36 ± 0.65^*^
Ethanolamine	11.78 ± 1.47	13.26 ± 1.80	13.42 ± 1.80	13.51 ± 1.64	13.22 ± 0.98	12.18 ± 1.96	13.37 ± 1.31	12.97 ± 1.47

a *Inoculated with water*.

b *Inoculated with FG*.

c *Mean ± SD, μmol/g freeze-dried plants*.

* *p < 0.05*,

** *p < 0.01 compared with wild-type*.

### Quantitative real-time PCR analysis

Total RNAs were isolated from aerial materials challenged by FG using Trizol reagent (Invitrogen) and treated with DNase I (Thermo) to remove contaminated genomic DNA. The first strand cDNA was prepared using superscript III reverse-transcriptase (Invitrogen). qRT-PCR analysis was performed on an iQ5 Cycler (Bio-Rad) under the previous condition (Chen et al., 2011) with three biological repeats. Two-tailed *t*-test (confidence interval, 95%) was performed using GraphPad Prism 5 software for the statistical analysis of the data. Gene-specific primers were used for the qRT-PCRs as listed in Table S2.

### Identification of the insertion sites of transgenic *A. thaliana* plants

Three pairs of nested primers (Table S3) were designed, based on the left arm sequence of the transformation vector pTRAKC, for use in Tail-PCR amplification according to the previous method (Liu and Chen, 2007). The insertion sites for Chi, CWP2 and Chi-CWP2 were chromosome 1 BAC F9K20, chromosome 5 BAC clone F2I11 and chromosome 2 clone F1O11 map g6825, respectively.

## Results

### Phenotypes of the *F. graminearum* infected *A. thaliana*

**Figure 1:**
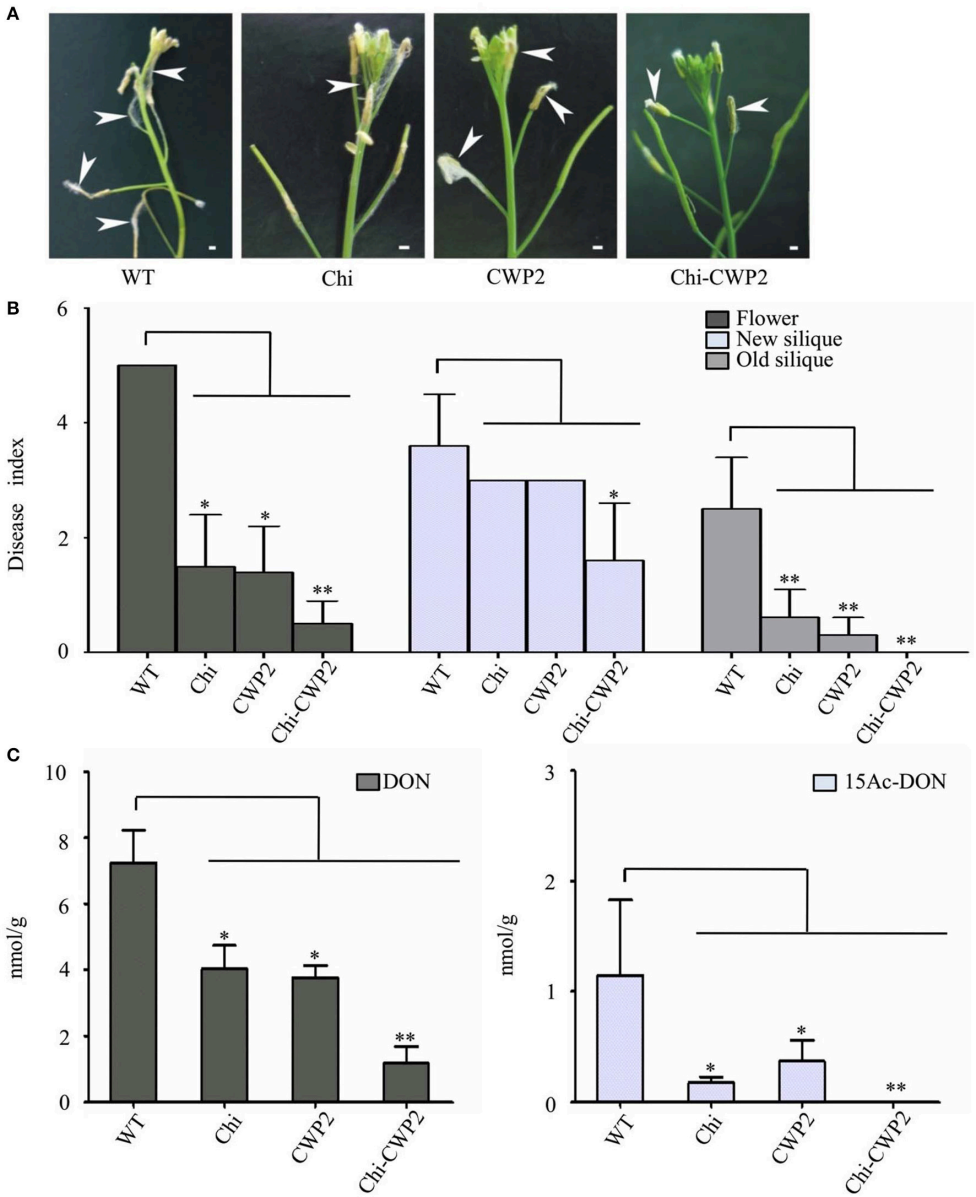
Phenotypes and mycotoxin production of *A. thaliana* on day-7 after FG inoculation. **(A)** Macroscopic features for floral tissue of *A. thaliana* (bar:1 mm); **(B)** disease indices and **(C)** levels of mycotoxins in floral tissues of wild-type (WT) and transgenic *A. thaliana* expressing Chi, CWP2 and Chi-CWP2 (SD from *n* = 30, ^*^p < 0.05, ^**^p < 0.01 in comparison with WT plants).

FG conidia were sprayed only into flowers to localize the infection, in this study, and all plants were maintained in the same growth chamber after inoculation until maturation. Therefore, FG-induced changes in plant morphological phenotypes were readily observable for flower but not any other tissues. For wild type *A. thaliana*, aerial mycelia were developed around anther and new siliques on day-3 post inoculation (3-DPI) of *F. graminearum* conidia. For *A. thaliana* expressing either Chi or CWP2, aerial mycelia were developed around anther and new siliques on 4-DPI but around flower and older siliques only after 7-DPI. *A. thaliana* expressing Chi-CWP2, in contrast, had significantly alleviation in such aerial mycelia development even after 7-DPI (Figure 1A).

On 7-DPI, the disease indices (DI) for flower and old siliques of all three transgenic plants expressing Chi, CWP2, and Chi-CWP2 respectively were significantly lower than that of wild type (Figure 1B). For new siliques, however, only Chi-CWP2 plants had significantly lower DI values than wild type (Figure 1B). Harvested ripen seeds from diseased plants were shriveled and dark brown in color whereas the healthy ones were plump and yellow in color (Figure S1). Compared to these from the uninfected plants, seeds from the FG-inoculated wild-type plants were markedly affected whereas about 50% seeds from Chi or CWP2 plants were affected. In contrast, most of the seeds from Chi-CWP2 plants were healthy after FG inoculation (Figure S1). All these data indicated that transgenic *A. thaliana* expressing a single gene Chi or CWP2 showed moderate FG resistance whereas, in contrast, the plant expressing fusion protein Chi-CWP2 displayed remarkably higher FG resistance (Figures 1A,B).

### Mycotoxins in flowers of the *F. graminearum* infected *A. thaliana*

It is well known that *F. graminearum* (FG) produces type-B trichothecene mycotoxins which typically include deoxynivalenol (DON) and its two derivatives 15-acetyl-deoxynivalenol (15Ac-DON) and 3-acetyl-deoxynivalenol (3Ac-DON); 15Ac-DON is much more toxic to plants than DON (Desjardins et al., 1993; Kimura et al., 2007). The levels of these mycotoxins positively associate with severity of FG-caused FHB in wheat (Gautam and Dill-Macky, 2011). Therefore, the levels of these mycotoxins in plant tissues generally have inverse correlation with FG resistance of the given plants. In this study, mycotoxins were measured for the FG-invaded flower tissues on 7-DPI (Figure 1C). DON levels in Chi and CWP2 plants (about 4.04 and 3.77 nmol per gram dry tissue, respectively) were much lower than in wild type (about 7.23 nmol/g); 15Ac-DON levels in Chi and CWP2 plants (0.18 and 0.37 nmol/g, respectively) were also significantly lower than that in wild type (1.14 nmol/g). In Chi-CWP2 plants, remarkably, DON level (1.18 nmol/g) was <50% of that in the other two transgenic lines;15Ac-DON level in Chi-CWP2 plants was close to detection limit and <10% of that in the other two transgenic lines (Figure 1C).

### Metabolomic phenotypes of FG-resistant transgenic *A. thaliana*

**Figure 2:**
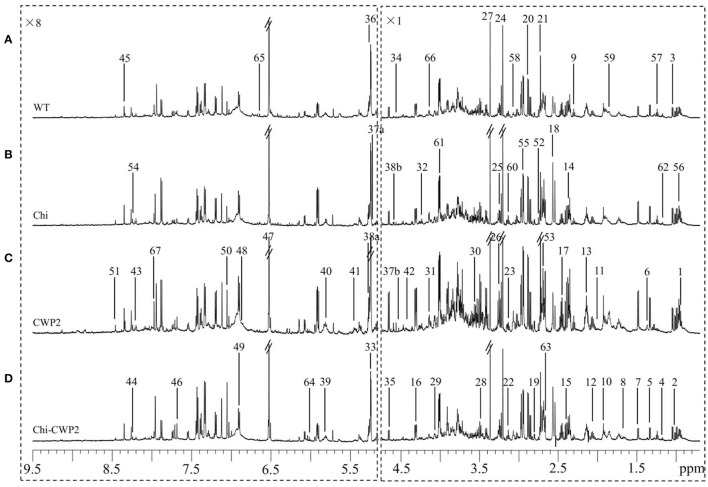
Typical ^1^H NMR spectra (600 MHz) of metabolite extracts from **(A)** wild-type (WT) and transgenic *A. thaliana* expressing **(B)** Chi, **(C)** CWP2, and **(D)** Chi-CWP2 after FG challenge. The spectra region δ 9.50–5.25 was vertically expanded 8 times. Metabolite keys: 1, leucine (Leu); 2, isoleucine (Ile); 3, valine (Val); 4, ethanol; 5, threonine (Thr); 6, lysine (Lys); 7, alanine (Ala); 8, arginine (Arg); 9, γ-aminobutyrate (GABA); 10, acetate; 11, proline (Pro); 12, glutamate (Glu); 13, glutamine (Gln); 14, pyruvate (Pyr); 15, succinate (Succ); 16, malate (Mal); 17, α-ketoglutarate (α-KG); 18, citrate (Cit); 19, aspartate (Asp); 20, asparagine (Asn); 21, dimethylamine; 22, ethanolamine (EA); 23, phenylalanine (Phe); 24, choline (Cho); 25, phosphocholine (PC); 26, betaine; 27, methanol; 28, tryptophan (Trp); 29, *myo*-inositol (mIno); 30, glycine (Gly); 31, fructose (Fru); 32, sucrose (Suc); 33, maltose; 34, N-methylnicotinate; 35, β-glucose (β-Glc); 36, α-glucose (α-Glc); 37a, α-arabinose (α-Arab); 37b, β-arabinose (β-Arab); 38a, α-galactose (α-Gal); 38b, β-galactose (β-Gal); 39, uridine (Uri); 40, uracil (Ura); 41, raffinose; 42, guanosine (Guan); 43, inosine (Ino); 44, hypoxanthine (Hyp); 45, adenosine (Aden); 46, sinapate; 47, fumarate (Fum); 48, polyphenolics; 49, tyrosine (Tyr); 50, histidine (His); 51, formate (Form); 52, sarcosine; 53, methionine (Met); 54, NAD^+^; 55, dimethylglycine; 56, α-aminobutyrate; 57, ethylmalonate; 58, α-ketoisovalerate; 59–67, unknown 1–9.

To reveal the plant metabolism associated with FG resistance, the FG-induced plant metabolomic changes were analyzed using NMR for the wild type and three transgenic *A. thaliana* lines expressing Chi, CWP2, and Chi-CWP2, respectively. ^1^H NMR spectra of all these plants showed rich information of metabolites (Figure 2) with more than 50 metabolites identified using the in-house databases and literature data (Fan, 1996; Fan and Lane, 2008; Xiao et al., 2009; Dai et al., 2010b). These included 20 amino acids (Leu, Ile, Val, Ala, Thr, Lys, Arg, Glu, Gln, Asp, Asn, Met, Pro, Phe, Trp, Tyr, His, Gly, D-α-aminobutyrate, and γ-aminobutyrate or GABA), 11 organic acids (pyruvate, succinate, malate, α-ketoglutarate, citrate, fumarate, formate, acetate, sinapate, ethylmalonate, and α-ketoisovalerate), 11 sugars (raffinose, sucrose, maltose, *myo*-inositol, β-glucose, α-glucose, α-arabinose, β-arabinose, α-galactose, β-galactose, and fructose), 8 nucleotide derivatives (NAD^+^, NMNA, uridine, uracil, guanosine, inosine, hypoxanthine, and adenosine), 6 choline metabolites (phosphocholine, choline, dimethylamine, dimethylglycine, sarcosine, and betaine) and some other metabolites (ethanol and ethanolamine). Such identification was further unambiguously confirmed with a series of 2D NMR spectra to obtain information of both ^1^H and ^13^C signals and their atomic connectivity (Table S4). Visual inspection of the NMR spectra revealed that the FG-infected transgenic plants expressing Chi-CWP2 contained higher levels of Phe, Trp, and His than the corresponding wild type (Figure 2).

From these spectral data, the contents of 30 metabolites were calculated from their non-overlapping signals (as μmol per gram freeze-dried tissue) for all four plant lines challenged with water and FG (Table 1). The results showed that glucose, Asn, Gln, Thr, citrate, and malate were the most abundant metabolites in these plants (Table 1). Without FG infection, transgenic lines showed some limited differences from the wild-type. Upon FG, however, such differences became much greater and more widespread metabolically. For instance, CWP2 plants contained about 58% more citrate, 40% more Phe and glucose, 20% more Thr and proline but 16–18% less GABA and Ala than the wild type (Table 1). In contrast, Chi-CWP2 plants contained about 110% more Phe and citrate, 37–48% more Trp, Tyr, His, citrate, glucose, *myo*-inositol, and betaine but 33% less Ala, 20% less choline and GABA than the wild type (Table 1). Chi-CWP2 plants also contained about 57% more Phe, 26–39% more *myo*-inositol, glucose, citrate, Tyr, and His, 18–26% more Trp and betaine but 13–15% less Ala and choline than Chi plants (Table 1).

Multiple univariate data analysis (MUDA) (Duan et al., 2013) was performed on all detectable metabolites (Figures S2, S3) to obtain more details in the differences of the plant metabolomic responses to fungal infection between different *A. thaliana* lines. *P*-values for all the metabolites having significant inter-group differences were obtained between the transgenic and wild-type *A. thaliana* sprayed with water and with FG (Table 2). Both Chi and CWP2 plants had only limited metabolic differences from the wild type when challenged with only water (without pathogens). For instance, Chi plants contained more glucose, fumarate and betaine but less *myo*-inositol, Arg and His than wild type; CWP2 plants contained more Asn and fumarate than wild type; Chi-CWP2 plants contained more Phe, Trp, His, Asn, pyruvate, and citrate than wild type (Table 2, Figure S2). It is interesting to note that Chi-CWP2 plants contain significantly more *myo*-inositol, Phe, Trp, His, Arg, Asn, and citrate but less fumarate than Chi and CWP2 plants (Table S5, Figure S2).

**Table 2:** *P*-values for inter-group differentiated metabolites in *A. thaliana*.

**Metabolites**	**Chi vs. WT^a^**	**CWP2 vs. WT^a^**	**Chi-CWP2 vs. WT^a^**	**Chi vs. WT^b^**	**CWP2 vs. WT^b^**	**Chi-CWP2 vs. WT^b^**
**SUGARS**						
*myo*-inositol	0.038^c^				0.038	0.038
α-glucose	0.032				0.028	0.015
**AMINO ACIDS**						
Thr					0.028	
Ala					0.022	0.011
Arg	0.038					
Pro					0.016	0.012
Asn		0.025	0.018			0.038
Phe			0.021	0.031	0.035	0.018
Trp			0.028			0.021
Tyr						0.028
His	0.004		0.028			0.025
GABA				0.032	0.035	0.038
**ORGANIC ACIDS**						
Pyruvate			0.025			0.018
Succinate						0.035
Malate				0.035	0.038	0.011
α-KG					0.038	0.012
Citrate			0.032	0.022	0.038	0.012
Fumarate	0.041	0.038				0.028
**NUCLEOSIDE/TIDES**						
Uridine						0.025
**CHOLINE METABOLITES**						
Choline						0.017
Betaine	0.038				0.017	0.038

a *Inoculated with water*.

b *Inoculated with FG*.

c *Red and blue numbers denote elevation and decrease of metabolites compared with the wild-type (WT), respectively. Only those with p < 0.05 were tabulated*.

After challenging with FG, however, the aforementioned inter-group metabolomic differences became much greater and metabolically more widespread. After FG challenge, Chi plants contained significantly more Phe, malate, and citrate but less GABA than wild type; CWP2 plants had significantly more glucose, *myo*-inositol, betaine, malate, α-ketoglutarate, citrate, Phe, Pro, and Thr but less GABA and Ala than wild type (Table 2, Figure S3). In contrast, Chi-CWP2 plants differed significantly from the other lines in their metabolic responses toward FG infection (Table S5). This transgenic line contained significantly more glucose, *myo*-inositol, Pro, Phe, Trp, Tyr, His, Asn, pyruvate, succinate, malate, α-ketoglutarate, citrate, fumarate, betaine, and uridine but less Ala, GABA, and choline than wild type (Table 2). Furthermore, Chi-CWP2 plants had significantly higher levels in glucose, betaine, Phe, Tyr, Trp, His, Arg, pyruvate, malate, α-ketoglutarate, and citrate but lower choline level than Chi plants (Table S5, Figure S3); Chi-CWP2 plants also had significantly higher levels in betaine, Phe, Tyr, Trp, His, pyruvate, malate, α-ketoglutarate, and citrate but lower levels in Ala, Thr, and choline than CWP2 plants (Table S5, Figure S3). These results were broadly consistent with the quantitative results (Table 1) though more comprehensive.

### Quantitative RT-PCR analysis for genes related to the altered metabolites

**Figure 3:**
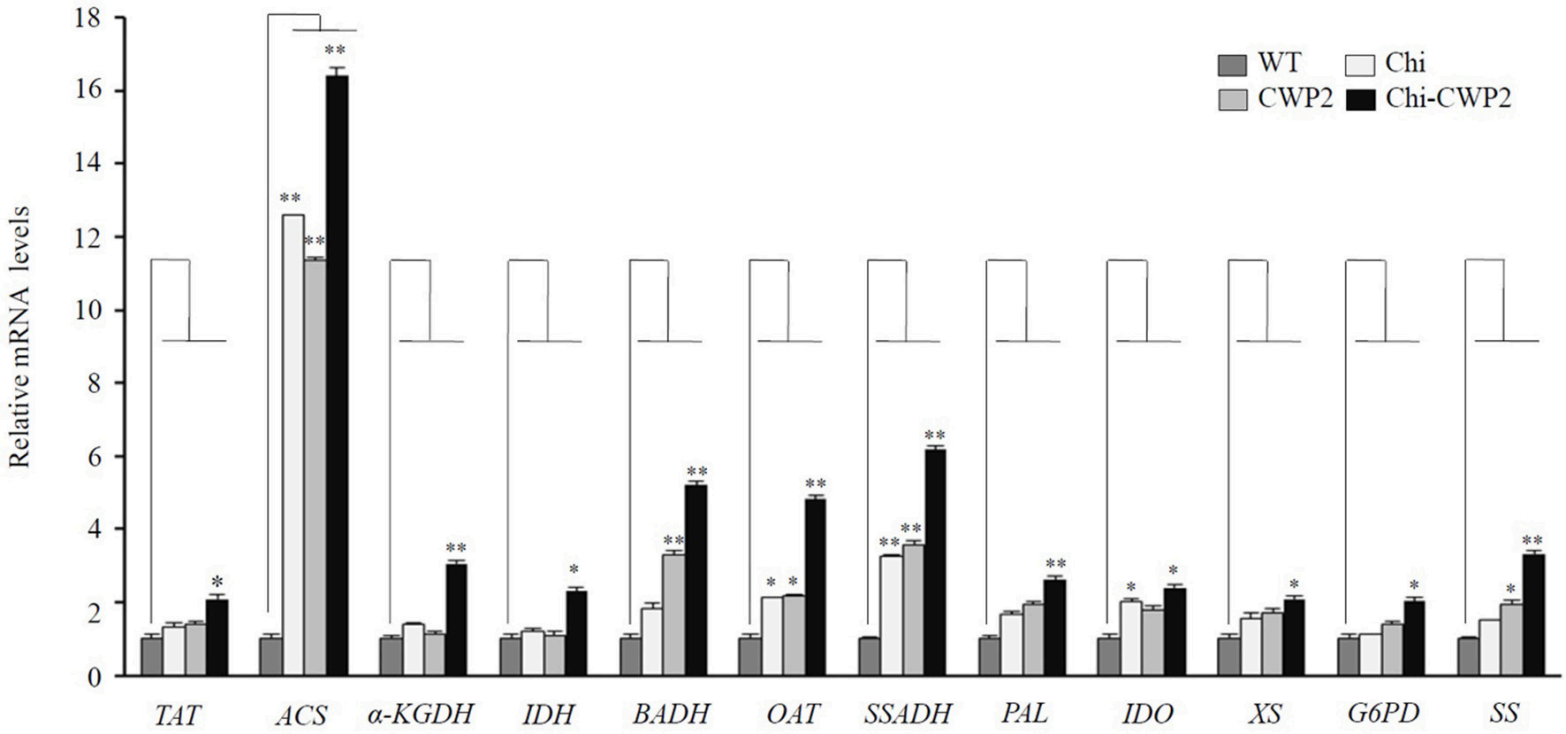
FG-induced expression changes measured by quantitative RT-PCR for some key genes from wild-type (WT) and transgenic *A. thaliana* expressing Chi, CWP2, and Chi-CWP2. Data were in the form of means ± *SD* (^*^P < 0.05; ^**^P < 0.01). *TAT*, tyrosine aminotransferase; *ACS*, acetyl-CoA synthetase; α*-KGDH*, α-ketoglutarate dehydrogenase; *IDH*, isocitrate dehydrogenase; *BADH*, betainealdehyde dehydrogenase; *OAT*, ornithine aminotransferase; *SSADH*, succinic-semialdehyde dehydrogenase; *XS*, xylan synthase; *G6PD*, glucose-6-phosphate-1-dehydrogenase; *IDO*, indoleamine 2,3-dioxygenase; *PAL*, phenylalanine ammonia-lyase; *SS*, strictosidine synthase.

To obtain information on some key genes regulating the metabolic pathways of the above altered metabolites, quantitative real-time PCR (qRT-PCR) analysis was performed with cDNA reverse-transcribed from mRNA isolated from four lines of *A. thaliana* after FG challenge. These included tyrosine aminotransferase (*TAT*) for biosynthesis of Phe and Tyr, ornithine aminotransferase (*OAT*) for proline biosynthesis, betaine-aldehyde dehydrogenase (*BADH*) for choline-to-betaine conversion, α-ketoglutarate dehydrogenase (α*-KGDH*) and isocitrate dehydrogenase (*IDH*) in TCA cycle, succinic-semialdehyde dehydrogenase (*SSADH*) in GABA shunt and acetyl-CoA synthetase (*ACS*) for acetate-to-acetyl-CoA conversion, phenylalanine ammonia-lyase (*PAL*) for phenylalanine to cinnamic acid conversion, indoleamine 2,3-dioxygenase (*IDO*) for tryptophan metabolism, xylan synthase (*XS*) for cell wall polysaccharide synthesis, glucose-6-phosphate-1-dehydrogenase (*G6PD*) for 6-phosphogluconate biosynthesis and strictosidine synthase (*SS*) for alkaloid biosynthesis. Compared to wild-type, FG inoculation led to significantly up-regulation of only *OAT*, S*SADH*, and *IDO* in Chi plants whereas up-regulation of *BADH, OAT, SSADH*, and *SS* in CWP2 plants (Figure 3). In contrast, all these seven genes in Chi-CWP2 were significantly up-regulated with FG inoculation; *ACS* was significantly up-regulated (more than 10-folds) in all three transgenic plants though more so in Chi-CWP2 plants (Figure 3).

**Figure 4:**
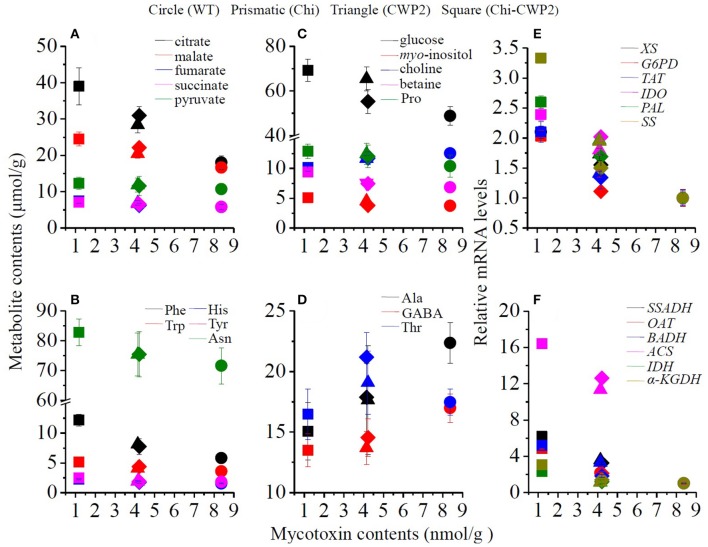
Correlations of mycotoxin levels with metabolites **(A–D)** and mRNA levels of some relevant key genes **(E,F)** for wild-type (dots) and transgenic *A. thaliana* expressing Chi (diamonds), CWP2 (triangles), and Chi-CWP2 (squares) after FG challenge.

Comprehensive correlations were observed between mycotoxins and some plant metabolites when collectively considering all data for the above four lines of *A. thaliana* (Figure 4). Production of mycotoxins was inversely correlated with the levels of aromatic amino acids (Phe, Tyr, Trp, His), osmolytes (proline, betaine, *myo*-inositol, glucose), TCA intermediates (citrate, succinate, fumurate, malate), Asn and pyruvate but positively correlated with the levels of choline, GABA and Ala (Figures 4A–D). Furthermore, the levels of mycotoxins were also inversely correlated with the expression levels of *TAT, BADH, OAT, IDH*, α*-KGDH, SSADH, ACS, PAL, IDO, XS, G6PD*, and *SS* (Figures 4E,F).

## Discussion

Transgenic plants expressing anti-*Fusarium* peptides and proteins have shown *Fusarium* resistance though natural germplasm has limited resistance. Chi has shown anti-fungal activity and FHB controlling potentials in birch and wheat (Punja, 2001; Pasonen et al., 2004; Shin et al., 2008) probably due to Chi's hydrolytic activity for chitin, which is a main cell wall polysaccharide in fungi but not in plants (Roncero, 2002; Ruiz-Herrera et al., 2002; Kim et al., 2009; Xu et al., 2010). Transgenic wheat expressing CWP2, a Fusarium-specific recombinant antibody, also showed some *Fusarium* resistance (Peschen et al., 2004; Cheng et al., 2015) whilst the CWP2 antibody fused to Chi showed excellent *Fusarium hyphae* inhibitory activity *in vitro* (Peschen et al., 2004). However, the roles of transgene-facilitated plant metabolic reprogramming in fungal resistance development remain to be fully understood. Therefore, this study investigated the FG resistance of *A. thaliana* expressing Chi, CWP2, and fused Chi-CWP2, in comparison with the wild-type line. Metabonomic responses of *A. thaliana* toward transgenesis and all these plants toward FG infection were systematically analyzed.

First, results from both disease index and mycotoxin production indicated that *Arabidopsis* plants expressing Chi, CWP2, and Chi-CWP2 had significantly better FG resistance than the wild-type (Figure 1). Whilst no significant differences in FG resistance were observed between Chi and CWP2 plants, Chi-CWP2 plants displayed remarkably higher resistance against FG than both Chi and CWP2 lines (Figure 1). The infected *Arabidopsis* flowers contained much more DON than 15Ac-DON (about 10% of total toxins) in all *A. thaliana* lines. Nevertheless, reduction of 15Ac-DON level is important for FG resistance since 15Ac-DON is more toxic to plants than DON (Kimura et al., 2006). After FG inoculation, noticeably, Chi-CWP2 plants contained significantly less DON than Chi and CWP2 plants with almost no 15Ac-DON in Chi-CWP2 plants (Figure 1C).

Metabolomic analysis showed that, under normal circumstances, plants expressing Chi and CWP2 had limited impacts to plant metabolism with elevation of fumurate (TCA cycle intermediate) and enhanced biosynthesis of osmolytes (betaine or glucose) compared with wild type (Tables 1, 2). Expression of Chi-CWP2 led to more metabolic alterations in *A. thaliana* than wild-type which was highlighted by significant elevation of aromatic amino acids (Phe, Trp, and His), citrate and Asn (Tables 1, 2). This probably indicates that expressing Chi-CWP2 mainly promotes the shikimate pathway-mediated secondary metabolism especially biosynthesis of phenylalanine-mediated phenylpropanoids and tryptophan-mediated alkanoids in addition to TCA cycle.

**Figure 5:**
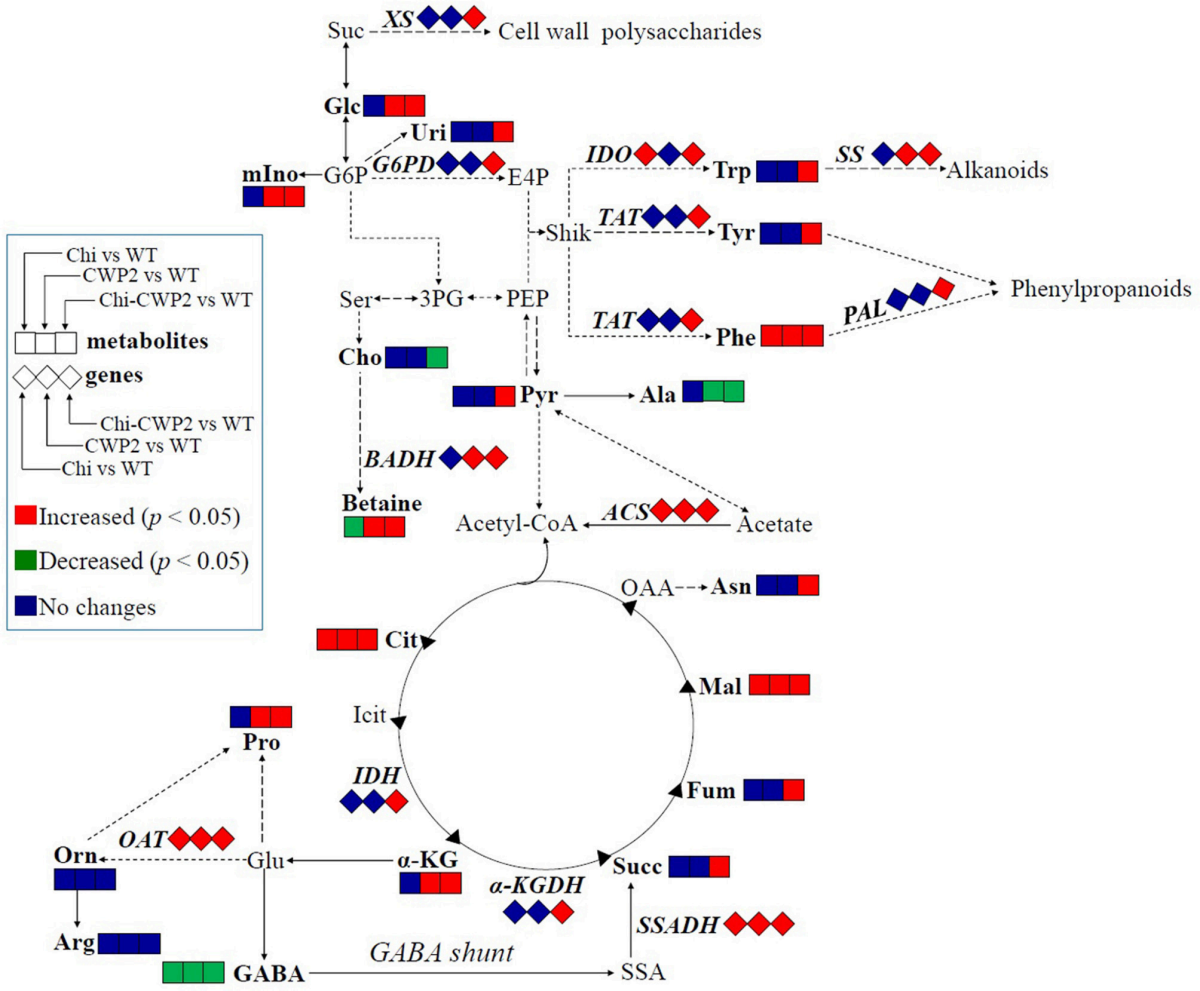
FG-induced metabolic changes in wild-type (WT) and transgenic *A. thaliana* expressing Chi, CWP2, and Chi-CWP2 highlighted by changes in metabolites and gene expression levels. Identified metabolites and measured genes were in bold letters. Red and green symbols denoted significant increases and decreases (*p* < 0.05), respectively. Suc, sucrose; Ala, alanine; Arg, arginine; Asn, asparagine; α-KG, α-ketoglutarate; Cho, choline; Cit, citrate; E4P, erythrose-4-phosphate; Fum, fumarate; GABA, γ-aminobutyrate; G6P, glucose-6-phosphate; Glc, glucose; Glu, glutamate; Icit, isocitrate; Mal, malate; mIno, *myo*-inositol; OAA, oxalacetate; Orn, ornithine; PEP, phosphoenolpyruvate; 3PG, 3-phosphoglycerate; Pyr, pyruvate; SSA, succinate semialdehyde; Ser, serine; Shik, shikimate; Succ, succinate; Trp, tryptophan; Tyr, tyrosine; Phe, phenylalanine; Uri, uridine.

When challenged with FG, however, more metabolic differences were observed between three transgenic plant lines and wild type as well as between transgenic plants having different FG resistance (Tables 1, 2, Figure 5). Compared to wild type, Chi transgenic plants having limited FG resistance showed elevation of Phe, malate, and citrate but decline of GABA (Tables 1, 2) suggesting enhanced shikimate-mediated secondary metabolism, TCA cycle, and probably GABA-shunt (Figure 5). CWP2 plants having moderate FG resistance (slightly better than Chi plants) showed elevation of Phe, malate, α-ketoglutarate, citrate, betaine, glucose, *myo*-inositol, and proline but decline of GABA and alanine (Tables 1, 2) indicating enhanced shikimate-mediated secondary metabolism, TCA cycle, biosynthesis of osmolytes, and GABA-shunt (Figure 5). This notion is further supported by moderate up-regulations of *BADH* for choline-mediated betaine biosynthesis, *SSADH* for GABA-to-succinate conversion in GABA shunt, *OAT* for ornithine-mediated proline biosynthesis and *SS* for alkaloid biosynthesis (Figures 1C, 5).

In contrast, however, plants expressing Chi-CWP2 with strong FG resistance had much more widespread and significant metabolic responses to FG challenge than wild type, Chi and CWP2 plants (Tables 1, 2, Figure 5). FG-induced elevation of phenylalanine, tyrosine and tryptophan (Tables 1, 2) together with up-regulation of *TAT, IDO, SS, G6PD*, and *PAL* (Figure 3) indicates promotion of the shikimate-mediated biosynthesis of phenylpropanoids and indole-related alkanoids (Figure 5). Several studies also supported the notion that both PAL and the phenylpropanoid pathway were involved in the resistance of wheat to FG and DON (Paranidharan et al., 2008; Walter et al., 2010). Elevations of succinate, malate, α-ketoglutarate, citrate, and fumarate (Tables 1, 2) together with up-regulations of α*-KGDH* and *IDH* (Figure 3) indicate enhancement of TCA cycle (Figure 5). Elevation of glucose, *myo*-inositol, pyruvate and concurrent decline of alanine suggest promotion of gluconeogenesis to generate glucose and *myo*-inositol as osmolytes (Liu et al., 2010; Kumar et al., 2016). Significant decline of GABA and up-regulation of *SSADH* suggest the promotion of GABA shunt to yield succinate feeding into TCA (Figure 5). Significant elevation of betaine, reduction of choline and up-regulation of *BADH* indicate promoted conversion of choline into betaine, which is a strong organic osmolyte (Figure 5). FG-induced elevation of proline and up-regulation of *OAT* probably suggests enhanced proline biosynthesis since ornithine aminotransferase catalyzes the conversion of ornithine into glutamate-5-semialdehyde and cyclization into 1-pyrroline-5-carboxylic acid followed with its reduction into proline. In *A. thaliana*, nevertheless, ornithine can also be turned into GABA *via* Arg-mediated putrescine-spermidine pathway, one cannot rule out such possibility here with the up-regulation of *OAT* especially with no changes in Arg and ornithine. The resultant GABA can be converted into succinate feeding into TCA cycle as discussed earlier and gluconeogenesis (Figure 5). This notion is further supported by elevation of Asn (*via* oxaloacetate-Asp route) and greatly enhanced *ACS* regulation probably to convert acetyl-CoA generated from TCA into acetate and then into pyruvate for gluconeogenesis (Figure 5).

The above propositions were further confirmed with strong correlations between these changed metabolites, gene expressions and production of mycotoxins (Figure 4). Inverse correlations between the levels of both mycotoxins and the expression levels of *TAT, ACS, IDH*, α*-KGDH, BADH, SSADH, OAT, PAL, IDO, XS, G6PD*, and *SS* (Figures 4E,F, Figure S4) suggest the above discussed metabolic reprogramming be positively associated with FG resistance. Inverse correlations between the production of mycotoxins and the levels of aromatic amino acids, TCA intermediates, osmolytes but positive correlation with the levels of choline and GABA (Figures 4A–D, Figure S4) suggest positive correlation of FG resistance with promoted biosynthesis of phenylpropanoids, indole-related alkanoids, osymolytes, and GABA shunt. This is conceivable since Phe, Tyr, and Trp are precursors for biosynthesis of phenylpropanoids and tryptophan-related alkanoids which have antibacterial activity in plant resistance to biotic stressors (Abdel-Farid et al., 2009). Biosynthesis of osmolytes such as glucose, *myo*-inositol, betaine, and proline are important metabolic reprogramming strategy for plant resistance to both abiotic and biotic stressors (Choi et al., 2004; Browne and Brindle, 2007; Paranidharan et al., 2008; Bollina et al., 2010; Dai et al., 2010b; Liu et al., 2010, 2017; Zhang et al., 2011; Kumar et al., 2016).

## Conclusion

Transgenic *A. thaliana* expressing Chi-CWP2 had much stronger FG resistance than wild-type and transgenic plants expressing Chi and CWP2 alone, both of which showed moderate FG resistance. Such FG resistance was highlighted in both reduced disease indices and production of mycotoxins with Chi-CWP2 plants producing much less mycotoxins after FG inoculation. The combined metabonomic and quantitative RT-PCR analyses revealed that enhanced biosynthesis of phenylpropanoids, tryptophan-related alkaloids, and organic osmolytes played important roles in FHB resistance for these transgenic *A. thaliana* lines especially Chi-CWP2 plants. Nevertheless, introduction of these genes had only limited metabolic effects on plants under normal circumstances thus limited effects on plant physiological biochemistry. These findings suggest that expressing Chi-CWP2 is an attractive approach for developing transgenic plants with intrinsic FG resistance. Concurrent enhancement of biosynthesis of shikimate-mediated secondary metabolites and organic osmolytes is a potentially important strategy worth exploring in development of the FG resistant plants in sustainable agriculture.

## Author contributions

Y-CL and HT: designed the project; FC, CL, and JZ: performed the sampling; FC and CL: measured the contents of mycotoxins and performed quantitative real-Time PCR analysis; FC, CL, and JZ: performed NMR measurements, spectral processing and data analysis; HL and H-PL: involved in the manuscript refinement; FC, CL, Y-CL, and HT: wrote the article.

### Conflict of interest statement

The authors declare that the research was conducted in the absence of any commercial or financial relationships that could be construed as a potential conflict of interest.
