# Supplementary material for: Combined Metabonomic and Quantitative RT-PCR Analyses Revealed Metabolic Reprogramming Associated with Fusarium graminearum Resistance in Transgenic Arabidopsis thaliana
Source: Front Plant Sci. 2018 Jan 4;8:2177. doi: 10.3389/fpls.2017.02177 (PMC5758590; doi:10.3389/fpls.2017.02177)
Supplement: Supplementary file 1 [file DataSheet1.PDF]

Supporting information

Combined metabonomic and quantitative RT-PCR analyses revealed metabolic reprogramming associated with *Fusarium graminearum* resistance in transgenic *Arabidopsis thaliana*

Fangfang Chen[1,2#], Caixiang Liu[3#], Jingtao Zhang[3], Hehua Lei[3], He-Ping Li[2], Yu-Cai Liao[2*], Huiru Tang[3,4,*]

[1]CAS Key Laboratory of Plant Germplasm Enhancement and Specialty Agriculture, Wuhan Botanical Garden, Chinese Academy of Sciences, Wuhan 430074, China.

[2]College of Plant Science and Technology and College of Life Science and Technology, Huazhong Agricultural University, Wuhan, 430070, China.

[3]CAS Key Laboratory of Magnetic Resonance in Biological Systems, State Key Laboratory of Magnetic Resonance and Atomic and Molecular Physics, National Centre for Magnetic Resonance in Wuhan, Wuhan Institute of Physics and Mathematics, Chinese Academy of Sciences, Wuhan, 430071, China.

[4]State Key Laboratory of Genetic Engineering, Zhongshan Hospital and School of Life Sciences, Collaborative Innovation Centre for Genetics and Development, Shanghai International Centre for Molecular Phenomics, Metabonomics and Systems Biology Laboratory, Fudan University, Shanghai, 200433, China.

[*]Correspondence should be addressed to H.T. (Huiru_tang@fudan.edu.cn) or Y.C.L. (yucailiao@mail.hzau.edu.cn)

[#] These authors contributed equally to this work.

Running title: Metabolic reprogramming of *A. thaliana* against FG

List of Supplementary Figures and Tables:

Figure S1. Phenotype for mature seeds of the *F. graminearum* inoculated *A. thaliana*.

Figure S2. Differential metabograms for wild-type (WT) and transgenic *A. thaliana* expressing Chi, CWP2 and Chi-CWP2, respectively, treated with water (but without FG).

Figure S3. Differential metabograms for wild-type (WT) and transgenic *A. thaliana* expressing Chi, CWP2 and Chi-CWP2, respectively, with FG challenge.

Figure S4. Heatmaps showing correlations of mycotoxin contents with the FG-induced significant changes of metabolites (a) and mRNA levels of twelve genes (b) for wild-type (WT) and transgenic *A. thaliana* expressing Chi, CWP2 and Chi-CWP2 respectively.

Table S1. T_1 values (seconds) for protons of some selected metabolites in *A. thaliana*.

Table S2. Primers for quantitative real-time PCR (qRT-PCR) analysis of gene expressions.

Table S3. Tail-PCR primers used in this study.

Table S4. NMR data and signal assignments for metabolites in wild-type and transgenic *A. thaliana* expressing Chi, CWP2 and Chi-CWP2.

Table S5. P-values for inter-group differentiated metabolites in transgenic *A. thaliana* expressing Chi, CWP2 and Chi-CWP2 inoculated with water and FG, respectively.

Figure S1. Phenotype for mature seeds of the *F. graminearum* inoculated *A. thaliana*.

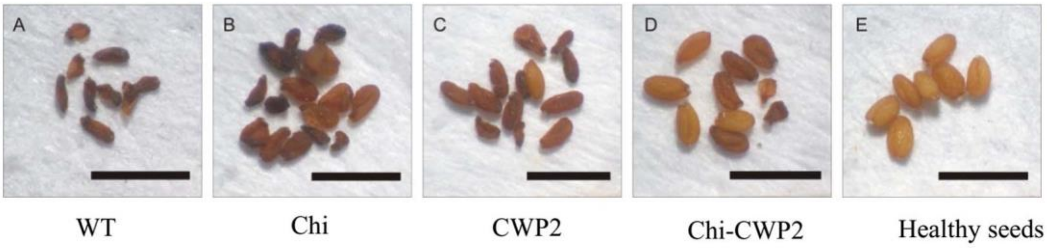

A, WT; B, Chi; C, CWP2; D, Chi-CWP2; E, Healthy seeds; Bar=1 mm.

Figure S2. Differential metabograms for wild-type (WT) and transgenic *A. thaliana* expressing Chi, CWP2 and Chi-CWP2, respectively, treated with water (but without FG). Only the red-colored metabolites had significant inter-group differences. Keys for metabolites are given in Table S4.

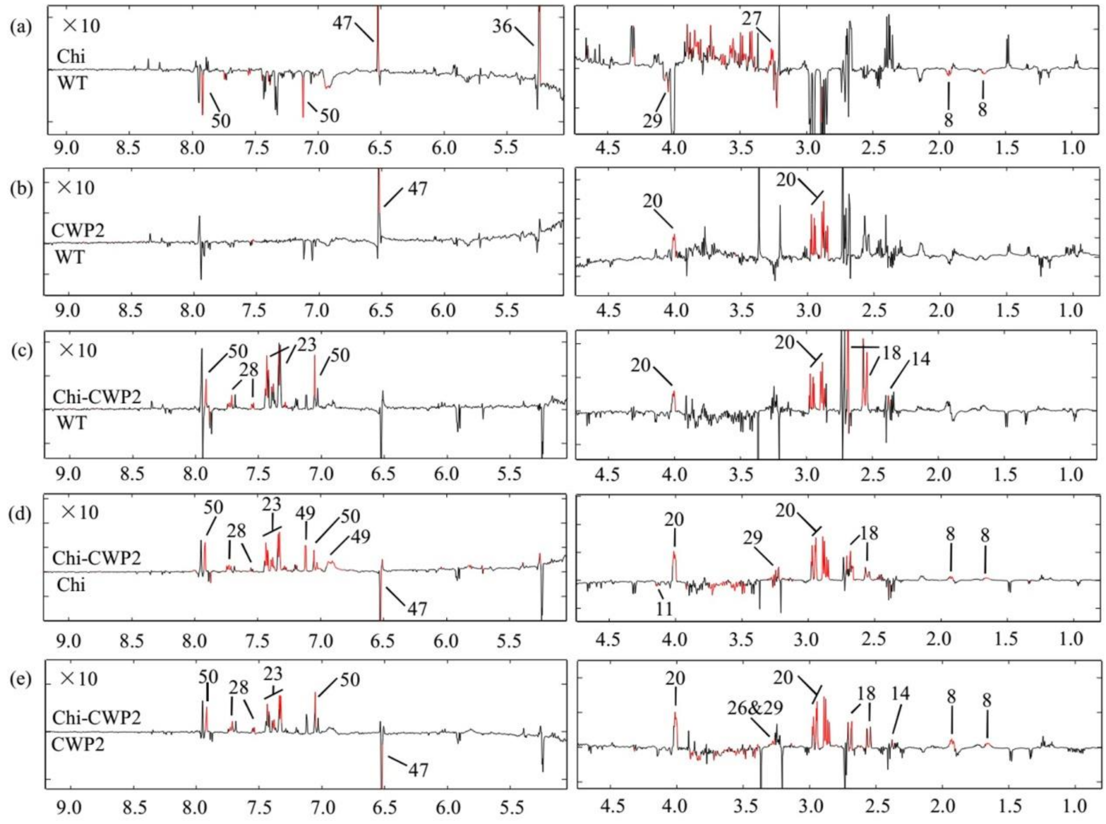

Figure S3. Differential metabograms for wild-type (WT) and transgenic *A. thaliana* expressing Chi, CWP2 and Chi-CWP2, respectively, with FG challenge. Only the red-colored metabolites had significant inter-group differences. Keys for metabolites are given in Table S4.

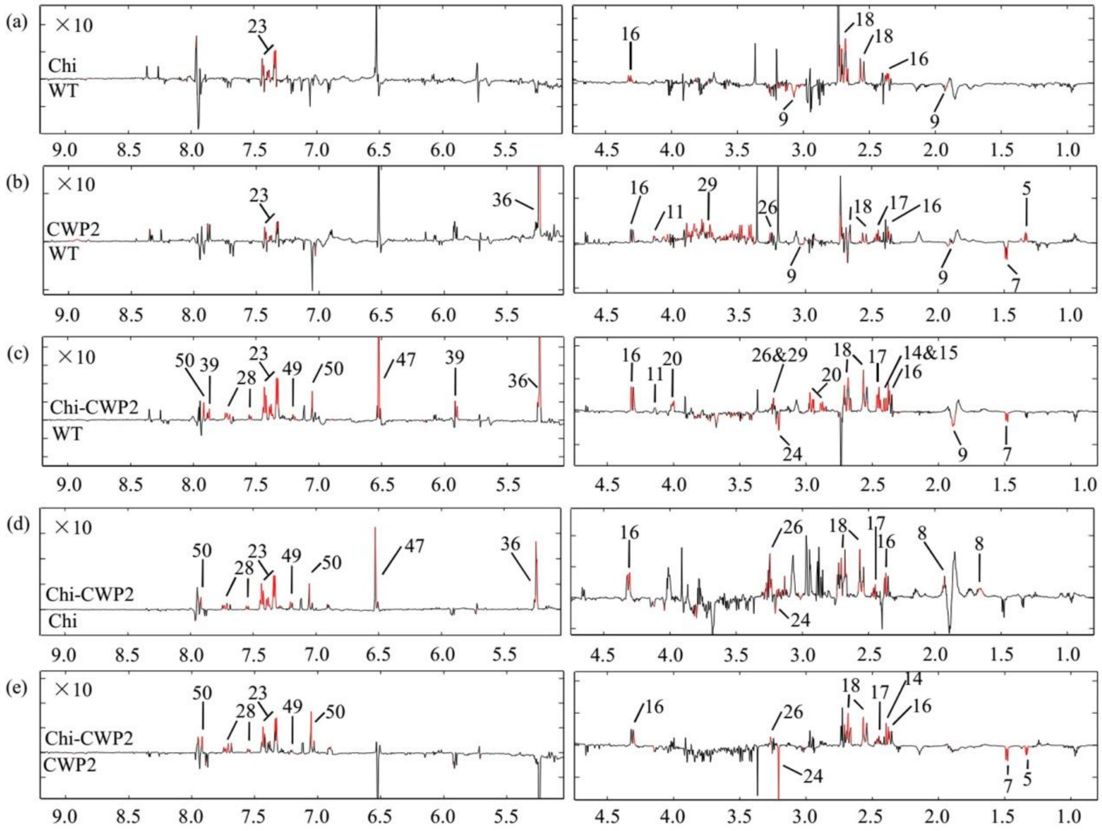

Figure S4. Heatmaps showing correlations of mycotoxin contents with the FG-induced significant changes of metabolites (a) and mRNA levels of seven genes (b) for wild-type (WT) and transgenic *A. thaliana* expressing Chi, CWP2 and Chi-CWP2 respectively. The correlation coefficients were color-coded with hot color (e.g., red) denoting positive correlations whereas the cool one (e.g., green) indicating negative ones. *TAT*, tyr aminotransferase; *ACS*, acetyl-CoA synthetase; *α-KGDH*, α-ketoglutarate dehydrogenase; *IDH*, isocitrate dehydrogenase; *BADH*, betaine-aldehyde dehydrogenase; *OAT*, ornithine aminotransferase; *SSADH*, succinic-semialdehyde dehydrogenase; *XS*, xylan synthase; *G6PD*, glucose-6-phosphate -1-dehydrogenase; *IDO*, indoleamine 2,3-dioxygenase; *PAL*, phenylalanine ammonia-lyase; *SS*, strictosidine synthase.

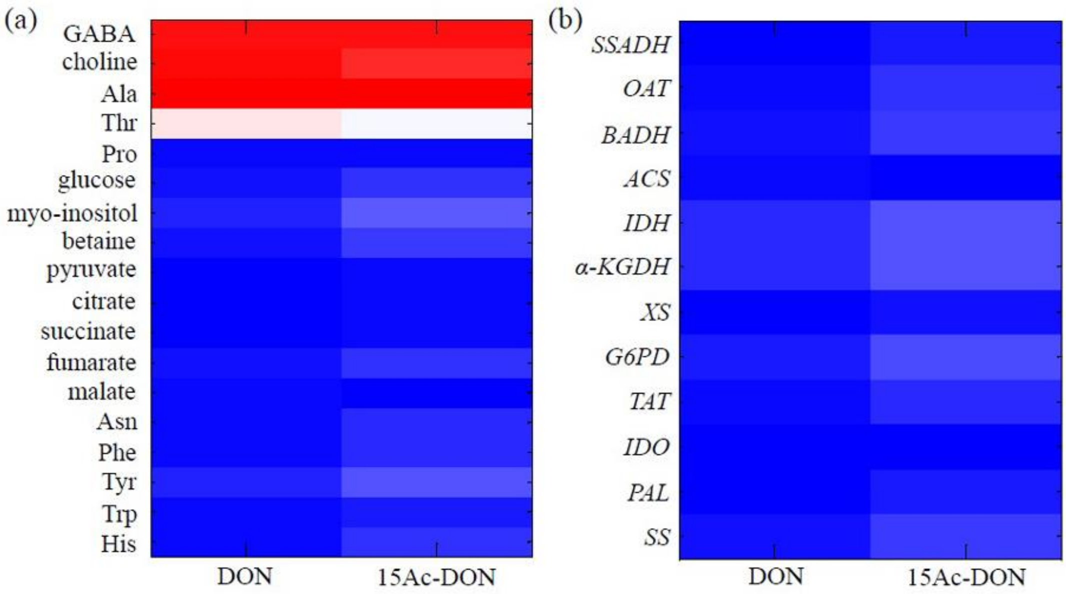

Table S1. T_1 values (seconds) for protons of some selected metabolites in *A. thaliana*.

metabolites	δ (multiplets)[a]	T_1 Values	
		Mean	SD
Tsp	0.00 (s)	2.03	0.01
alanine	1.48 (d)	2.42	0.01
isoleucine	1.01 (d)	0.85	0.01
valine	1.04 (d)	1.01	0.01
threonine	1.33 (d)	0.94	0.01
γ-aminobutyrate	2.30 (t)	1.47	0.01
pyruvate	2.37 (s)	1.46	0.06
succinate	2.41 (s)	1.07	0.01
glutamine	2.45 (m)	1.22	0.01
citrate	2.56 (d)	0.18	0.02
aspartate	2.80 (dd)	0.63	0.01
asparagine	2.88 (dd)	1.13	0.01
ethanolamine	3.14 (t)	2.42	0.02
choline	3.19 (s)	2.33	0.01
betaine	3.26 (s)	1.51	0.02
myo-insitol	3.27 (t)	1.51	0.01
fructose	4.11 (d)	1.07	0.01
proline	4.14 (m)	ND	ND
malate	4.32 (dd)	0.25	0.01
galactose	5.26 (d)	1.93	0.02
α-glucose	5.23 (d)	2.42	0.01
sucrose	5.40 (d)	1.07	0.01
fumarate	6.52 (s)	1.98	0.01
histidine	7.11 (s)	1.70	0.01
phenylalanine	7.42 (m)	2.50	0.01
trytophan	7.53 (d)	2.09	0.02
tyrosine	6.90 (d)	2.59	0.02
uridine	7.85 (d)	1.48	0.01
inosine	8.22 (s)	ND	ND
hypoxanthine	8.24 (s)	2.70	0.01
adenosine	8.32 (s)	1.27	0.01

ND: T_1 value was not determined because of signal overlapping; [a] δ: chemical shifts.

Table S2. Primers for quantitative real-time PCR (qRT-PCR) analysis of gene expressions.

	Gene encoded protein	Sequence (F: Forward, 5'-3'; R: Reverse, 5'-3')	Gene
1	beta-tubulin	F: TGCTATTCTGCGTTTGGACCTTG<br>R: ATCCCTTACGATTTCACGCTCTG	At3g12110
2	tyrosine aminotransferase (TAT)	F: GGGAATGTTTTCTCTCGCCAA<br>R: CACCATCCAACCAAGTCTCCA	At5g36160
3	acetyl-CoA synthetase (ACS)	F: TAAACGCCGTCGTGTTAGGAGA<br>R: TATCAGACCAAAAAGCAGCAGGG	At5g36880
4	α-ketoglutarate dehydrogenase(α-KGDH)	F: TCTTCTTCTGCTTCGGGATTGG<br>R: TGGACACCCTGGACAAGAACG	At5g55070
5	isocitrate dehydrogenase subunit (IDH)	F: ACTGCTGCTGGTATTGCTGGA<br>R: TGCTCTTCCGTTGTATCCTTCC	At2g17130
6	betaine-aldehyde dehydrogenase(BADH)	F: CCACCGAGGATGAGGCAATT<br>R: GCCTGGAAAGCCTTACTAACACG	At3g48170
7	ornithine aminotransferase (OAT)	F: ATGATGCTACTCGTGGATTCGG<br>R: TTTCAAGTAACCGTCGGGAGG	At5g46180
8	succinic-semialdehyde dehydrogenase (SSADH)	F: GCGGCAACGCACCCTCTAT<br>R: CCCATCCCTAAACCCATCTCCT	At1g79440
9	phenylalanine ammonia-lyase (PAL)	F:AAGTGAAGAGAATGGTTGCTGAG<br>R:GAAGTGCGACACCGTTTTTG	At2g37040
10	indoleamine 2,3-dioxygenase (IDO)	F: CACAACACCCACAACTCCAAAAG<br>R: TATGCCAAATCCAACTGCCACTG	At4g02610
11	xylan synthase (XS)	F: AACGAGCAGTTCTGGGTCATAGG<br>R: CGCAAACTCATCGTCTCCATCT	At1g02730
12	glucose-6-phosphate -1-dehydrogenase (G6PD)	F: GAAGGACGTGGAGGGTACTTTG<br>R: TCGGTGTAGGCAGGATATGTGA	At5g13110
13	strictosidine synthase (SS)	F:TTTGTCTCGTTGATTTCTCTTCTCC<br>R:CGTTAGTGGTTCCAAGTGCTCC	At1g74000

Table S3. Tail-PCR primers used in this study.

Primers	Sequence (5'-3')
LAD1	ACGATGGACTCCAGAGCGGCCGC (G /C /A) N (G /C /A) NNNGGAA
LAD2	ACGATGGACTCCAGAGCGGCCGC (G /C /T) N (G /C /T) NNNGGTT
LAD3	ACGATGGACTCCAGAGCGGCCGC (G /C /A) (G /C /A) N (G /C /A) NNNCCAA
LAD4	ACGATGGACTCCAGAGCGGCCGC (G /C /T) (G /A /T) N (G /C /T) NNNCGGT
AC	ACGATGGACTCCAGAG
LB0a	CGCGCGATATAAAACAAAAGATAGCTCATAA
LB1a	ACGATGGACTCCAGTCCGGCCTTAAATAGGATCAAACGCG
LB2a	ATGGTCTATAGTCGAACGTACGGCC

Table S4. NMR data and signal assignments for metabolites in wild-type and transgenic *A. thaliana* expressing Chi, CWP2 and Chi-CWP2.

NO	metabolites	moiety	δ^1 H (multiplicity)[a]	δ^{13} C	experiments
1	Leucine (Leu)	δ-CH_3	0.94 (t, 7.2 Hz)	24.4	TOCSY, HSQC,
		δ′-CH_3	0.96 (t, 6.3 Hz)	26.3	HMBC
		γ-CH_2	1.69 (m)	26.4	
		β-CH_2	1.72 (m)	42.6	
		α-CH	3.69 (t, 8.7 Hz)	56.9	
2	Isoleucine (Ile)	δCH_3	0.94 (t, 7.2 Hz)	16.2	TOCSY, HSQC,
		β-CH_3	1.01 (d, 7.0 Hz)	18.9	HMBC
		γ-CH_2	1.25 (m)	27.1	
		β-CH	1.98 (m)	38.4	
		α-CH	3.65 (m)	63.3	
3	Valine (Val)	γ-CH_3	1.04 (d, 7.2 Hz)	20.4	TOCSY, HSQC,
		γ′-CH_3	0.99 (d, 7.0 Hz）	19.2	HMBC
		β-CH	2.27 (m)	31.8	
		α-CH	3.61 (d, 5.0 Hz)	63.3	
		COOH	\	175.6	
4	Ethanol	CH3	1.17 (t, 6.1 Hz)	19.4	TOCSY, HSQC,
		CH2	3.66 (q, 2.0 Hz)	60.1	HMBC
5	Threonine (Thr)	CH_3	1.33 (d, 6.6 Hz)	21.9	TOCSY, HSQC,
		α-CH	3.59 (q, 4.7 Hz)	63.2	HMBC
		β-CH	4.27 (m)	68.7	
		COOH	\	185.1	
6	Lysine (Lys)	γ-CH_2	1.45, 1.51 (m)	24.2	TOCSY, HSQC,
		δ-CH_2	1.73 (m)	26.6	HMBC
		β-CH_2	1.91 (m)	30.2	
		ε-CH_2	3.03 (t, 7.5 Hz)	43.1	
		α-CH	3.76 (t, 5.0 Hz)	56.9	
7	Alanine (Ala)	CH_3	1.48 (d, 7.3 Hz)	18.9	TOCSY, HSQC,
		CH	3.79 (q, 6.3 Hz)	53.6	HMBC
		COOH	\	178.7	
8	Arginine (Arg)	γ-CH	1.68, 1.72 (m)	26.5	TOCSY, HSQC,
		β-CH2	1.91 (m)	30.0	HMBC
		δ-CH2	3.25 (m)	43.1	
		α-CH	3.78 (t, 4.7 Hz)	56.9	
9	γ-aminobutyrate (GABA)	β-CH_2	1.91 (m)	26.3	TOCSY, HSQC,
		α-CH_2	2.30 (t, 7.4 Hz)	36.7	HMBC
		γ-CH2	3.02 (t, 7.9 Hz)	42.2	
		COOH	\	184.3	
10	Acetate (Acet)	CH_3	1.92 (s)	26.7	HSQC, HMBC
		COOH	\	177.3	
11	Proline (Pro)	γ-CH_2	2.01 (m)	26.6	TOCSY, HSQC,
		β-CH_2	2.35 (m)	29.6	HMBC

		δ-CH_2	3.34, 3.41 (m)	48.7	
		α-CH_2	4.14 (m)	63.5	
		COOH	\	177.6	
12	Glutamate (Glu)	β-CH_2	2.02, 2.09 (m)	29.4	TOCSY, HSQC,
		γ-CH_2	2.36 (m)	35.9	HMBC
		α-CH	3.75 (m)	57.4	
		COOH	\	182.5	
		COOH	\	177.7	
13	Glutamine (Gln)	β-CH_2	2.14 (m)	29.1	TOCSY, HSQC,
		γ-CH_2	2.45 (m)	33.5	HMBC
		α-CH	3.75 (m)	56.9	
		COOH	\	208.2	
		C=O	\	184.1	
14	Pyruvate (Pyr)	CH_3	2.37 (s)	29.5	HSQC
15	Succinate (Succ)	CH_3	2.41 (s)	36.2	HSQC
		COOH	\	183.4	
16	Malate (Mal)	β'-CH	2.37 (dd, 10.1, 15.3 Hz)	45.9	TOCSY, HSQC, HMBC
		β-CH	2.68 (dd, 3.1, 15.4 Hz)	45.9	
		CH	4.31 (dd, 3.0, 10.1 Hz)	72.6	
		COOH	\	183.9	
17	α-ketoglutarate (α-KG)	γ-CH_2	2.45 (t, 7.9 Hz)	35.9	TOCSY, HSQC
		β-CH_2	3.02 (t, 8.3 Hz)	41.6	
18	Citrate (Cit)	1/2CH_2	2.55 (d, 15.8 Hz）	51.5	TOCSY, HSQC,
		1/2CH_2	2.68 (d, 15.8 Hz）	51.5	HMBC
		3 C	\	78.3	
		COOH	\	177.3	
		COOH	\	180.4	
19	Aspartate (Asp)	β-CH_2	2.68 (dd,7.6, 16.5 Hz)	39.0	TOCSY, HSQC,
		β'-CH_2	2.80 (dd,4.4, 17.5 Hz)	39.0	HMBC
		α-CH	3.89 (dd, 4.3, 7.6 Hz)	55.0	
		COOH	\	176.9	
20	Asparagine (Asn)	β-CH	2.87 (dd,7.6, 16.5 Hz)	37.1	TOCSY, HSQC,
		β'-CH	2.95 (dd,4.6, 16.5 Hz)	37.1	HMBC
		α-CH	4.00 (dd, 4.6, 7.6 Hz)	54.0	
		γ-$CONH_2$	\	177.1	
		COOH	\	176.9	
21	Dimethylamine	CH_3	2.72(s)	39.2	HSQC
22	Ethanolamine (EA)	N-CH_2	3.15 (t, 5.2 Hz)	44.0	TOCSY, HSQC,
		O-CH_2	3.83 (t, 5.1 Hz)	60.1	HMBC
23	Phenylalanine (Phe)	β-CH_2	3.12 (dd,7.8, 14.9 Hz), 3.25 (dd, 5.3, 14.7 Hz)	38.9	TOCSY, HSQC, HMBC
		α-CH	4.00 (m)	58.9	
		3,5CH,	7.33 (m)	131.8	

		ring			
		3,5CH, ring	7.38 (m)	130.2	
		3,5CH, ring	7.43 (m)	131.7	
		COOH	\	174.9	
24	Choline (Cho)	N-$(CH_3)_3$	3.20 (s)	56.6	TOCSY, HSQC, HMBC
		N-CH_2	3.52 (m)	\	
		O-CH_2	4.07 (m)	70.1	
25	Phosphocholine (PC)	N-$(CH_3)_3$	3.23 (s)	56.6	TOCSY, HSQC
		N-CH_2	4.23 (m)	\	
		O-CH_2	3.61 (m)	\	
26	Betaine	CH_3	3.26 (s)	56.6	TOCSY, HSQC, HMBC
		CH_2	3.91 (s)	72.4	
		COOH	\	174.6	
27	Methanol	CH_3	3.36 (s)	51.7	TOCSY, HSQC
28	Tryptophan (Trp)	β-CH_2	3.31 (dd,4.9, 14.3 Hz), 3.48 (dd, 9.1, 14.0 Hz)	27.9	TOCSY, HSQC, HMBC
		α-CH	4.05 (m)	58.4	
		6 CH, ring	7.29 (t, 7.3 Hz)	120.1	
		7 CH, ring	7.53 (d, 8.2 Hz)	114.6	
		4 CH	7.74 (d, 8.0 Hz)	120.7	
		COOH	\	175.8	
29	Myo-inositol (mIno)	2-CH	4.07 (t, 2.5 Hz)	75.1	TOCSY, HSQC, HMBC
		1,3-CH	3.52 (dd, 5.1, 10.9 Hz)	75.3	
		4,6-CH	3.61 (t, 4.9 Hz)	73.9	
		5-CH	3.29 (t, 4.5 Hz)	77.1	
30	Glycine (Gly)	CH_2	3.57 (s)	44.3	TOCSY, HSQC, HMBC
		COOH	\	175.3	
31	Fructose (Fru)	3 CH	3.79, 3.81 (m)	63.8	TOCSY, HSQC
		4 CH	4.01 (m)	54.1	
		3 CH	4.11 (d, 3.7 Hz)	77.8	
32	Sucrose (Suc)	F-1 CH	4.22 (d, 8.8 Hz)	94.8	TOCSY, HSQC
		G-1 CH	5.42 (d, 3.8 Hz)	75.2	
33	Maltose	2 CH	5.22 (d, 3.8 Hz)	102.7	TOCSY, HSQC
		11 CH	5.42 (d, 3.8 Hz)	102.1	
34	NMNA[b]	2 CH	9.13 (s)	148.4	TOCSY, HSQC
		6 CH	8.84 (t)	147.7	
		4 CH	8.85 (t)	145.8	
		CH_3	4.44 (s)	51.0	
35	β-glucose (β-Glc)	2 CH	3.25 (dd, 2.9, 7.0 Hz)	76.8	TOCSY, HSQC
		3 CH	3.49 (m)	78.6	
		4 CH	3.42 (m)	72.6	
		5 CH	3.47 (m)	78.7	

		6,6′ CH	3.73, 3.90 (m)	63.5	
		1 CH	4.65 (d, 8.0 Hz)	98.7	
36	α-glucose (α-Glc)	4 CH	3.42 (m)	72.2	TOCSY, HSQC
		2 CH	3.53 (dd, 3.8, 9.9 Hz)	74.1	
		3 CH	3.73 (m)	74.8	
		6,6′ CH	3.74, 3.83 (m)	63.5	
		5 CH	3.86 (m)	74.3	
		1 CH	5.23 (d, 3.7 Hz)	94.8	
37a	α-arabinose (α-Arab)	α-1 CH	5.21 (d, 4.0 Hz)	94.5	TOCSY, HSQC
		\	3.87 (m)	63.3	
37 b	β- arabinose (β-Arab)	β-1 CH	4.52 (d, 8.2 Hz)	98.8	TOCSY, HSQC
		\	3.52 (dd, 5.5, 10.2 Hz)	\	
		\	3.69 (m)	\	
38a	α-galactose (α-Galac)	1 CH_2	5.27 (d, 3.8 Hz)	94.9	TOCSY, HSQC
		2 CH	3.81 (dd, 4.7, 8.7 Hz)	73.6	
		3 CH	3.97 (m)		
38 b	β-galactose (β-Galac)	1 CH_2	4.59 (d, 7.7 Hz)	99.2	TOCSY, HSQC
		2 CH	3.49 (dd, 5.3, 10.2 Hz)	78.4	
		3 CH	3.67 (m)	\	
39	Uridine (Uri)	6 CH	4.36 (t, 4.9 Hz)	77.1	TOCSY, HSQC
		12 CH	5.90 (d, 8.1 Hz)	104.9	
		7 CH	5.92 (d, 4.5 Hz)	91.7	
		11 CH	7.88 (d, 8.1 Hz)	144.3	
40	Uracil (Ura)	5 CH	5.80 (d, 7.7 Hz)	103.6	TOCSY, HSQC
		6 CH	7.54 (d, 7.6 Hz)	147.4	
41	Raffinose	G-1 CH	5.44 (d, 3.7 Hz)	95.0	TOCSY, HSQC
		F-1 CH	4.99 (d, 3.6 Hz)	101.1	
		G-1 CH	3.57 (m)	74.1	
42	Guanosine (Guan)	CH	8.01 (s)	138.0	TOCSY, HMBC
		5’-CH_2	5.90 (d, 4.4 Hz)	91.9	
		4’-CH	4.41 (dd, 4.8, 10.4 Hz)	73.8	
		3’-CH	4.22 (m)	88.5	
43	Inosine (Ino)	2 CH	6.08 (d, 6.1 Hz)	92.8	HSQC
		7 CH	8.21 (s)	\	
		12 CH	8.32 (s)	143.3	
44	Hypoxanthine (Hyp)	2 CH	8.20 (s)	\	JRES, TOCSY
		7 CH	8.24 (s)	\	
45	Adenosine (Aden)	14 CH	8.35 (s)	143.2	TOCSY, HSQC
		8 CH	8.26 (s)	155.1	
		1 CH	6.06 (d, 5.7 Hz)	91.0	
46	Sinapate	8 CH	6.50 (d, 15.8 Hz)	117.9	TOCSY, HSQC, HMBC
		7 CH	7.67 (d, 15.8 Hz)	149.1	
		1 CH	\	128.5	
		2,6 CH	7.01 (s)	108.8	

		3,5 CH	\	139.7	
		4 CH	\	151.0	
		4-OCH_3	3.89 (s)	58.7	
		COOH	\	172.0	
47	Fumarate (Fum)	2,3 CH	6.52 (s)	138.0	TOCSY, HSQC,
		COOH	\	177.7	HMBC
48	Polyphenolics	\	6.89 (m)	108.9	TOCSY, HSQC,
		\	7.63 (m)	123.7	HMBC
49	Tyrosine (Tyr)	β-CH_2	3.05 (dd,7.7, 14.9 Hz), 3.15 (dd, 8.5, 14.4 Hz)	39.2	TOCSY, HSQC, HMBC
		α-CH	3.93 (m)	58.7	
		3,5 CH, ring	6.90 (d, 8.6 Hz)	118.3	
		2,6 CH, ring	7.20 (d, 8.4 Hz)	133.2	
		COOH	\	175.1	
50	Histidine (His)	4 CH, ring	7.09 (s)	119.8	TOCSY,HSQC,
		4 CH, ring	7.92 (s)	138.6	HMBC
		β-CH_2	3.20 (dd, 5.1, 14.7 Hz), 3.25 (dd, 9.4, 14.6 Hz)	30.5	
		α-CH	4.00 (m)	56.6	
		COOH	\	174.6	
51	Formate (Form)	CH	8.46 (s)	173.5	HSQC
52	Sarcosine	CH_3	2.75 (s)	38.9	TOCSY, HSQC,
		COOH	\	180.2	HMBC
53	Methionine (Met)	γ-CH2	2.65 (t, 7.5 Hz)	32.6	TOCSY, HSQC
		β-CH2	2.17 (m)	32.9	
		α-CH	3.78 (m)	56.8	
		S-CH3	2.14 (s)	16.1	
		COOH	\	174.3	
54	NAD^{+b}	N2 CH	9.47 (s)	143.6	TOCSY,HSQC,
		N4 CH	8.19 (t, 6.9 Hz)	130.5	HMBC
		N5 CH	9.16 (d, 6.2 Hz)	143.8	
		N6 CH	8.95 (d, 7.8 Hz)	149.4	
		N1' CH	6.23 (d, 4.8 Hz)	57.3	
		A2 CH	8.32 (s)	143.7	
		A6 CH	8.23 (s)	155. 0	
		A1' CH	6.06 (d, 5.6 Hz)	90.9	
55	Dimethylglycine	CH_3	2.94 (s)	54.6	TOCSY, HSQC
			\	41.2	
56	D-α-aminobutyrate[b]	2 CH_2	1.89 (m)	27.5	TOCSY, HSQC
		1 CH	3.68 (t, 5.8 Hz)	56.5	
		3 CH_3	0.97 (t, 7.4 Hz)	11.2	
		COOH	\	176.7	

57	Ethylmalonate[b]	5 CH_3	1.24 (t, 7.1 Hz)	15.3	TOCSY, HSQC,
		6 CH_2	3.28 (s)	42.6	HMBC
		4 CH_2	4.17 (q, 7.2 Hz)	63.1	
		2 COOH	\	169.2	
		7 COOH	\	171.3	
58	α-ketoisovalerate[b]	7,8 CH_3	1.10 (d, 6.7 Hz)	19.8	TOCSY, HSQC,
		6 CH	3.05 (dq, 7.1 Hz)	41.5	HMBC
		COOH	\	178.2	
		COOH	\	196.1	
59	U1	\	1.37	30.6	TOCSY, HSQC
		\	1.85 (m)	23.7	
		\	3.07	56.6	
60	U2	\	0.86 (d, 6.4 Hz)	\	JRES, TOCSY
		\	3.12 (s)	\	
		\	3.36 (m)	\	
		\	3.75 (m)	\	
		\	4.23 (m)	\	
61	U3	\	1.29 (d, 6.0 Hz)	19.5	JRES, TOCSY, HSQC
		\	3.55 (dd, 3.7, 11.1 Hz)	30.9	
		\	3.73 (dd, 5.1, 11.1 Hz)	\	
		\	3.96 (dd, 5.8, 12.5 Hz)	74.5	
		\	4.12 (d, 3.8 Hz)	77.5	
62	U4	\	1.14 (d, 6.1 Hz)	19.0	JRES, TOCSY,
		\	3.47 (m)	72.2	HSQC
		\	3.96 (dd, 5.8, 12.5 Hz)	74.3	
		\	4.15 (m)	\	
63	U5	\	2.65 (dd,11.5, 15.5 Hz)	42.3	JRES, TOCSY,
		\	5.16 (dd, 2.7, 11.2 Hz)	76.6	HSQC
64	U6	\	6.02 (d, 12.5 Hz)	119.6	JRES, TOCSY
		\	7.02 (d, 12.6 Hz)	147.2	
65	U7	\	6.43 (d, 15.8 Hz)	\	JER, TOCSY,
		\	6.64 (d, 15.8 Hz)	97.6	HSQC
66	U8	\	4.19 (dd, 2.5, 8.0 Hz)	78.7	JRES, TOCSY,
		\	4.61 (dd, 2.4, 5.3 Hz)	75.2	HSQC
67	U9	\	7.97 (s)	\	JRES, TOCSY

[a]Multiplicity: s, singlet; d, doublet; dd, doublet of doublets; t, triplet; q, quartet; m, multiplet; U, unidentified signal; \, signals or multiplicities were not determined; [b], tentatively assigned.

Table S5. P-values for inter-group differentiated metabolites in wild-type and transgenic *A. thaliana* expressing Chi, CWP2 and Chi-CWP2 inoculated with water and FG, respectively.

Metabolites	Chi-CWP2 *vs* Chi[a]	Chi-CWP2 *vs* CWP2[a]	**Chi-CWP2 *vs* Chi**[b]	**Chi-CWP2 *vs* CWP2**[b]
**Sugars**				
myo-inositol	0.038	0.038		
α-glucose			0.020	
**Amino acids**				
Thr				0.017
Ala				0.025
Arg	0.025	0.038	0.004	
Pro	0.025			
Asn	0.035	0.021		
Phe	0.025	0.023	0.035	0.032
Trp	0.025	0.032	0.023	0.019
Tyr	0.038		0.038	0.040
His	0.002	0.038	0.027	0.038
GABA				
**Organic acids**				
pyruvate		0.020		0.010
succinate				
malate			0.032	0.038
α-KG			0.028	0.025
citrate	0.028	0.035	0.029	0.027
fumarate	0.028	0.038	0.034	
**Nucleoside/tides**				
uridine				0.025
**Choline metabolites**				
choline			0.015	0.018
betaine		0.038	0.004	0.017

[a] inoculated with water; [b] inoculated with FG. [c] red and green signs denote elevation and decrease of metabolites, respectively. Only those with $p < 0.05$ were tabulated.
